# Relating Macroscopic PET Radiomics Features to Microscopic Tumor Phenotypes Using a Stochastic Mathematical Model of Cellular Metabolism and Proliferation

**DOI:** 10.3390/cancers16122215

**Published:** 2024-06-13

**Authors:** Hailey S. H. Ahn, Yas Oloumi Yazdi, Brennan J. Wadsworth, Kevin L. Bennewith, Arman Rahmim, Ivan S. Klyuzhin

**Affiliations:** 1 Department of Physics and Astronomy, University of British Columbia, Vancouver, BC V6T 1Z1, Canada; 2 Department of Integrative Oncology, BC Cancer Research Institute, Vancouver, BC V5Z 1L3, Canada; 3 Department of Pathology and Laboratory Medicine, University of British Columbia, Vancouver, BC V6T 1Z3, Canada

**Keywords:** radiomics, PET, metabolic heterogeneity, modeling, tumor phenotype

## Abstract

**Simple Summary:**

Radiomics analysis of positron emission tomography (PET) images can provide objective measurements of tumor heterogeneity and spatial patterns. However, the relatively low resolution, high noise, and limited longitudinal data availability make it difficult to systematically investigate the relationship between the microscopic tumor phenotypes and corresponding PET radiomics signatures. To address this challenge, we use a multiscale, stochastic mathematical model of tumor growth to generate cross-sections of tumors in vascularized normal tissue on a microscopic level. By varying the biological parameters of the model, distinct tumor phenotypes are obtained, and their corresponding PET images are generated. The simulated data are then used to find the optimal combination of PET radiomics features that can reliably distinguish visually similar tumor phenotypes. In addition, we study the longitudinal changes in the discriminative power of radiomics features with tumor growth from a single cell to approximately one million cells.

**Abstract:**

Cancers can manifest large variations in tumor phenotypes due to genetic and microenvironmental factors, which has motivated the development of quantitative radiomics-based image analysis with the aim to robustly classify tumor phenotypes in vivo. Positron emission tomography (PET) imaging can be particularly helpful in elucidating the metabolic profiles of tumors. However, the relatively low resolution, high noise, and limited PET data availability make it difficult to study the relationship between the microenvironment properties and metabolic tumor phenotype as seen on the images. Most of previously proposed digital PET phantoms of tumors are static, have an over-simplified morphology, and lack the link to cellular biology that ultimately governs the tumor evolution. In this work, we propose a novel method to investigate the relationship between microscopic tumor parameters and PET image characteristics based on the computational simulation of tumor growth. We use a hybrid, multiscale, stochastic mathematical model of cellular metabolism and proliferation to generate simulated cross-sections of tumors in vascularized normal tissue on a microscopic level. The generated longitudinal tumor growth sequences are converted to PET images with realistic resolution and noise. By changing the biological parameters of the model, such as the blood vessel density and conditions for necrosis, distinct tumor phenotypes can be obtained. The simulated cellular maps were compared to real histology slides of SiHa and WiDr xenografts imaged with Hoechst 33342 and pimonidazole. As an example application of the proposed method, we simulated six tumor phenotypes that contain various amounts of hypoxic and necrotic regions induced by a lack of oxygen and glucose, including phenotypes that are distinct on the microscopic level but visually similar in PET images. We computed 22 standardized Haralick texture features for each phenotype, and identified the features that could best discriminate the phenotypes with varying image noise levels. We demonstrated that “cluster shade” and “difference entropy” are the most effective and noise-resilient features for microscopic phenotype discrimination. Longitudinal analysis of the simulated tumor growth showed that radiomics analysis can be beneficial even in small lesions with a diameter of 3.5–4 resolution units, corresponding to 8.7–10.0 mm in modern PET scanners. Certain radiomics features were shown to change non-monotonically with tumor growth, which has implications for feature selection for tracking disease progression and therapy response.

## 1. Introduction

Cancer is classified as a set of diseases related to uncontrolled cell proliferation and is a leading cause of death globally [1]. Various hallmarks of cancer, including resistance to cell death, genetic diversity, vascular network reconstruction, and dynamic tumor tissue microenvironment, result in distinct tumor phenotypes with significant heterogeneity between and within tumors. An increasing amount of evidence is showing that more heterogeneous tumors tend to exhibit more aggressive progressions and are more resistant to treatment with conventional therapy types. Moreover, tumors with similar heterogeneous properties have shown similar progression patterns and sensitivity to treatment despite manifesting at different locations [2,3,4]. Despite the inherent variability in tumor phenotypes, even for the same type of cancer, the de facto standard of care follows the “one-size-fits-all” approach wherein a standard dose is delivered to most patients. However, it has been shown that such an approach only works well for 25% of patients [5]. The growing field of precision medicine aims to tackle this problem and make a shift toward personalized treatments, including a larger role of imaging and radiomics [6].

Given a sufficiently large tumor size, positron emission tomography (PET) imaging with tracer ^18^F-fluorodeoxyglucose (FDG), a glucose analog, can be used to assess the metabolic heterogeneity of tumors in vivo. Accurate and standardized quantification of radiomics features in PET images that describe tumor shape, texture, and morphology can provide meaningful information relating to tumor heterogeneity. For instance, in a tumor texture analysis study by Orlhac et al., several texture indices were highly correlated with the molecular volume across three tumor types [4]. Another study by Hatt et al. [3] showed that heterogeneity quantification in five different tumor types had prognostic value for clinical decisions. An increasing amount of evidence is showing that imaging-derived radiomics signatures can facilitate better cancer diagnosis, prognosis, and treatment planning that is not just specific to each patient, but to each distinct tumor [7].

Despite numerous research studies on leveraging PET radiomics in different cancer types, there is a lack of understanding of how the observable tumor heterogeneity in PET images, quantified via radiomics feature extraction, is linked to the properties of the tumor microenvironment. No models exist that establish a link between the tissue microenvironmental parameters and functional heterogeneity visible on PET images. Additionally, due to ethical, methodological, and economic reasons, there is a lack of longitudinal data showing how PET radiomics signatures of tumors change over time with tumor growth when no treatment intervention is applied.

To address these issues, here, we develop a new methodology to generate realistic simulated images of small tumors with a full longitudinal growth pattern. We employ a hybrid, multiscale mathematical model of tumor growth in vascularized tissue [8] to generate realistic spatial distributions of cells that are, in turn, converted to synthetic PET images showing glucose metabolism. The model is hybrid in the sense that it combines (1) grids of agents, namely autonomous, decision-making entities representing different cell types and blood vessels and (2) partial differential equation (PDE) grids to simulate nutrient diffusion and exchange. The model uses realistic biological parameters and constants, such as diffusion coefficients, cell sizes, cell cycle durations, and well-established metabolism models. We are able to generate continuous time-sequences of agent (cell) maps and convert them to PET parametric images of pseudo-standardized uptake values (pSUV), giving us the ability to study any PET radiomics feature as a function of model parameters and time.

The proposed methodology allows us to investigate the changes in radiomics features with tumor growth (longitudinal study) and small alterations in tumor phenotype (cross-sectional study). Below, we describe the proposed method in detail and list the values of the used model microparameters that we obtained from previous biological, biochemical, and histological studies. To demonstrate the utility of the method, we generated longitudinal synthetic PET images of six distinct tumor phenotypes obtained by varying the model’s microparameters. We used the simulated data to find the optimal set of radiomics features for phenotype differentiation and tumor progression tracking. We demonstrated that by using specific pairs of radiomics features, all six generated phenotypes can be numerically distinguished within 1 cm diameter tumors under realistic resolution and noise.

## 2. Methods

### 2.1. Tumor Growth Simulations

Simulations of tumor growth in vascularized tissue were carried out on a microscopic level using a previously proposed and validated hybrid multiscale mathematical model [8]. The model combines agents and partial differential equations (PDEs) to simulate tissue properties and individual cell behavior, which in turn depends on the local state of the tissue microenvironment. The unique advantage of the hybrid model is that any combination of agent grids and PDE grids can be used, allowing multiple layers of complexity (e.g., the model can incorporate multiple drugs and/or radionuclides). Furthermore, the components execute independently while the data are easily interfaced between the PDE grids, which enables varying spatial and temporal scales to describe the biological processes involved in solid tumor growth. This is key to modeling tumor growth, as tumor tissue is dynamic and complex, with continuous interactions with its microenvironment. The interplay between the agents and the PDE grids defines the tumor characteristics and progression patterns in the simulations, resulting in distinct tumor phenotypes.

**Figure 1:**
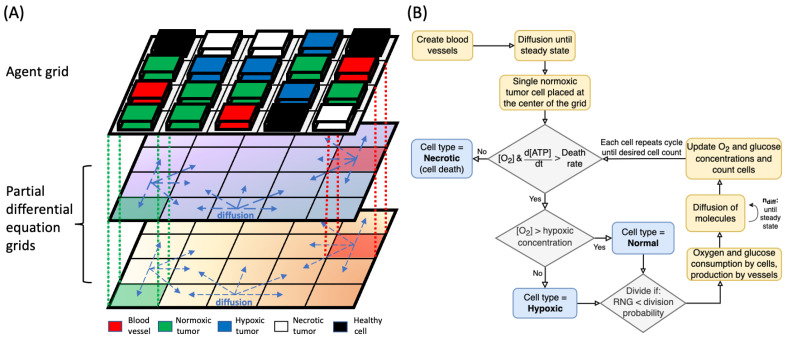
(**A**) Components of the hybrid mathematical model used in the simulation. The model uses a combination of one agent grid and two PDE grids for oxygen and glucose, as shown. The dashed vertical lines illustrate the link between the agent and PDE grids. Blocks of different color represent different types of agents, and arrows indicate diffusion processes in the PDE grids. Blood vessels are the sources of nutrients in the simulation. (**B**) Flowchart of the main algorithm for tumor growth simulation, including the steps of simulation initialization, cell type determination, and the processes involved in the change of nutrient concentrations. [O_2_] is the oxygen concentration at the cell location, and $n_{diff}$ is the number of diffusion steps required to reach steady state for each cell step.

The agent types in the model are blood vessels, normal (healthy) cells, and three different states of cancer cells categorized as normoxic, hypoxic, and necrotic (Figure 1A). Agents occupy identical sized pixels (20 × 20 μm) on a 2D grid, and they are unstackable, meaning that only one agent is allowed per pixel at a time. Blood vessels act as the source of nutrients such as oxygen and glucose that diffuse to surrounding tissues; oxygen and glucose concentrations in blood vessels were 50 mmHg and $5\times 10^{-3}$ mol/L, respectively. Cancerous and normal cells consume the nutrients to produce ATP, and the rate of ATP production determines the cell behavior and proliferation rate.

The PDE grids are used to (1) update the concentration of nutrients at each grid location in every simulation step and to (2) compute the concentration differentials resulting from the diffusion of molecules from blood vessels and consumption by cells. Diffusion of molecules in tissue follows Fick’s diffusion law with distinct diffusion coefficients (*D*) associated with each type of molecule:

(1) $$\frac{\partial C}{\partial t}=D\nabla^{2}C+f$$

where *C* is the local concentration, and *f* is the consumption rate specific to each molecule. The consumption rate of a molecule follows the Michaelis–Menten model that describes the enzyme kinetics of oxygen and glucose, given as follows:

(2) $$f=-V_{max}\times \frac{\left[S\right]}{K_{M}+\left[S\right]},$$

where $V_{max}$ is the maximum rate, [*S*] is the substrate concentration, and $K_{M}$ is the concentration at half maximum. Deviations in $V_{max}$ and $K_{M}$ reflect the upregulated metabolism in cancer cells compared to normal cells.

The oxygen consumption rate that is simplified to a constant value for each cell type as $K_{M}$ for oxygen in tissue is negligible compared to the oxygen concentration in tissue ($K_{M}\ll \left[S\right]$):

(3) $$f=-V_{max}\times \frac{\left[S\right]}{K_{M}+\left[S\right]}=-V_{max}$$

Knowing the consumption rates for oxygen and glucose, we can calculate the ATP production rates. Aerobic respiration yields ∼27 ATP/glucose, yet tumor cells with altered metabolism utilize glycolysis as the predominant pathway, producing 2 ATP/glucose. Thus, the total ATP production rate is given as follows:

(4) $$f_{ATP}=-\left(2f_{G}+\frac{27f_{O}}{5}\right).$$

The ATP production rate for each cell determines the actions available to that cell in every simulation step, as elaborated below.

### 2.2. Simulation Steps

Utilizing one of the advantages of the hybrid model, we used different temporal scales for the agent grid and the PDE grids. Each simulation step in the agent map corresponded to 1 biological hour, in which the individual cells determine their cell type and the action to be taken between each iteration. Diffusion of molecules, on the other hand, had a much smaller time scale of 30 ms between each step, which was restricted by the upper limit of the unitless diffusion coefficient in the model where the partial differential equations remain numerically stable. Thus, one cell simulation step included multiple diffusion steps; the number of diffusion steps was empirically set to be sufficiently large to reach a steady state in nutrient concentrations between each cell step.

At the beginning of each simulation, the agent grid was entirely occupied by normal cells and blood vessels (Figure 1B). Multiple iterations of the diffusion simulation were applied to allow nutrients to diffuse from blood vessels and reach steady concentrations throughout the PDE grids.

To initiate the tumor growth after a steady state in nutrient concentrations had been reached, we placed a single normoxic cancer cell at the center of the grid. Indeed, most tumors are presumed to originate from a single cell that has accumulated a critical number of genetic mutations through interactions with its microenvironment [9].

Next, the behavior of the cell in the simulation cycle was determined by the local oxygen and glucose concentrations from the corresponding location in the PDE grids. First, conditions were evaluated for cell survival. Cell death can occur in two ways: apoptosis and necrosis. Apoptosis refers to the programmed cell death that is necessary for discarding unwanted cells that include aged and defective cells. Necrosis, on the other hand, is induced by external factors such as nutrient deficiency and trauma. Since normal apoptosis is typically disrupted in cancer cells, our model only includes necrosis caused by deficiency in glucose and/or oxygen supply. Biologically, a cell can become necrotic when the oxygen concentration or the ATP production rate falls below the threshold levels (0.08% O_2_ and $42.82\times 10^{-17}$ mol ATP/s/cell) for a prolonged period of time [10,11]. To reflect this in the model, we introduce a variable cell death probability that depends on the threshold values of oxygen concentration and ATP production:

(5) $$1-{\left(\frac{local\;value}{threshold\;value}\right)}^{x}$$

where *x* is a variable power. When oxygen or ATP production levels fall below the threshold level and the random number sampled between 0–1 is less than the death probability, the cell is deemed necrotic. Once a cell enters the necrotic state, it does not consume any nutrients or go through further division.

After the survival determination, it was determined whether the cell should enter a hypoxic state. A cell was determined hypoxic if the oxygen concentration in the corresponding location on the PDE grid was in the range of 0.08–0.5% O_2_. In grid locations with O_2_ greater than 0.5%, hypoxic cells were converted to normoxic cells.

In the next step, if adequate nutrient supply was available, viable cells (normoxic and hypoxic) could divide into adjacent locations available in the Moore neighborhood of the agent grid, producing a daughter cell of the same type and kinetic parameters for metabolism. Available locations for producing a daughter cell included pixels occupied by normal cells and vessels; the vessel location is chosen only when there is no normal cell to replace in its neighborhood. Cell division probability was implemented so that a proliferating cell in normoxic conditions would on average divide once every 24 biological hours [12,13]. Other cells in a lower oxygenation environment had a division probability that scaled proportionally with the local oxygen concentration, with a maximum probability equal to that of normoxic cells. If the concentrations were over the threshold level and the random number generated fell within the division probability, the cell would undergo a division.

Using the above sequence, the state of all agents on the agent grid was updated in each simulation step. After each agent grid update, multiple diffusion simulation steps were taken to reach the new steady-state of nutrient concentration. Thus, from the “point of view” of the agent grid, the nutrient concentrations are always in a steady state, which is a reasonable assumption given the vastly different timescales of the molecular diffusion and cellular life cycles.

### 2.3. Model Parameters and Implementation

For the computation of nutrient supply, diffusion, and consumption rates by cells, the model used several biological parameters such as oxygen and glucose diffusion coefficient in tissue, cell size, and metabolic rate constants. A list of biological parameters that were kept constant in all models is shown in Table 1.

The pixel size used was 20 × 20 μm^2^, chosen based on a typical human cell size, and the 2D simulation grids were composed of 2000 × 2000 pixels, corresponding to a (4×4) cm^2^ square grid. To minimize edge effects coming from a finite grid size, we imposed periodic boundary conditions on the nutrient concentration PDE grids to simulate a section of a larger tissue.

### 2.4. Tested Model Alterations

Blood vessel density and spatial distribution in tissue play an essential role in shaping the local microenvironment and consequently the phenotype of a growing tumor. Thus, to simulate different tumor phenotypes, we adjusted the following tissue microparameters of the model: (a) blood vessel density, (b) vessel removal (obstruction) probability, (c) vessel distribution pattern, and (d) cell death probability from an insufficient ATP production rate. In total, six different phenotypes were simulated, with the respective parameter values given in Table 2.

Vessel removal probability is the probability that a dividing cancer cell replaces a vessel in its neighborhood, which happens only when all other locations are already occupied by cancer cells. This parameter characterizes the degree of vascular network alteration by tumor cells.

The blood vessel distribution pattern on the grid was either random or uniform with small deviations, depending on the simulated phenotype.

Necrotic death probability reflects the probability of necrosis due to prolonged oxygen or ATP depletion in the local tissue, and *R* is the ratio of local oxygen concentration or ATP production rate to a threshold value below which necrosis becomes possible. The model for necrotic probability due to oxygen depletion was 1−R for all simulations, but the corresponding probability model for ATP production rate was varied, as shown in Table 2. Varying this parameter changes the specific cell population’s resistance to external stress, in this case lack of nutrients.

Other parameters such as vascular nutrient concentration, diffusion coefficients in tissue, and maximum metabolic rates for each cell type were set to a fixed value in all simulations within a realistic physiological range (Table 1).

Five realizations of longitudinal growth were simulated for each phenotype. All simulations started with a single cancer cell placed at the center of the agent grid and continued until the tumors were approximately 1.7 cm in diameter. During the simulations, a snapshot of the agent grid every 10 h of biological time was exported as TIFF images, where pixel RGB values represented different cell types. The resulting 30 (6 phenotypes × 5 realizations) time series of images were used to generate synthetic PET images for radiomics analysis.

### 2.5. Preparation and Imaging of Tumor Xenografts

Simulated agent maps of tumors were compared to real histology images of human cancer cells grown in animal models. Animal experiments were performed as previously described [22,23]. Briefly, 10-week-old male NOD/SCID-gamma mice were housed under specific pathogen-free conditions. WiDr human colon adenocarcinoma and SiHa human cervical squamous cell carcinoma cell lines were maintained in minimal essential medium (MEM; Life Technologies) + 10% fetal bovine serum (Sigma, St. Louis, MO, USA) under standard culture conditions (37 °C, 5% CO_2_) and used within 25 passages. Flank tumors were produced by subcutaneous injection of 106 cells in 100 µL of filter-sterilized phosphate-buffered saline. Tumors were harvested 21–32 days post-implant when average tumor sizes were between 300 and 400 mg. All animal experiments were performed in accordance with institutional and Canadian Council on Animal Care guidelines.

Pimonidazole (100 mg/kg; Hypoxyprobe, Burlington, MA, USA) was administered by intraperitoneal injection 90 min prior to tumor harvest. Intravenous injection of Hoechst 33342 (1 mg in 50 µL) 10 min prior to mouse sacrifice was used to label cells surrounding perfused blood vessels [24]. Excised tumors were cut in half prior to being embedded in optimal cutting temperature medium (OCT; Tissue Tek) and frozen for subsequent sectioning, in which 10 µm thick tumor sections were cut from OCT-embedded tumors using a cryostat.

Pimonidazole was detected using a monoclonal antibody (Hypoxyprobe) conjugated to fluorescein isothiocyanate (FITC). Slides were stained for CD31 using rabbit monoclonal antibody (Pharmogen 553377) with Alexa Fluor 594 secondary antibody (Life Technologies, Carlsbad, CA, USA) to identify blood vessel endothelium.

The immunofluorescent microscopy image acquisition system consisted of a robotic fluorescence microscope (Zeiss AxioImager Z1, Carl Zeiss, Jena, Germany), a cooled, monochrome CCD camera (Retiga 4000R, QImaging, Surrey, BC, Canada), and a motorized slide loader and x-y stage (Ludl Electronic Products, Hawthorne, NY, USA). Images of whole tumor sections were obtained by tiling together individual fields of view with a resolution of 1.5 µm/pixel. Images were collected in monochrome for each fluorescence detection channel and were pseudocolored with blue or green after collection.

### 2.6. Conversion of Agent Maps to Synthetic PET Images

To convert agent maps to synthetic pSUV images, we made an approximation that for each cell, the tracer (presumed to be FDG) uptake depends only on the type and metabolic rate of the cell; thus, we neglected possible effects of restricted blood supply and endogenous-exogenous competition.

Assuming that normal healthy tissues have a pSUV of ∼1.0, the pSUVs for different states of cancer cells can be obtained from their relative glucose consumption rates. Oxygenated tumor cells show about an 8 times higher uptake than normal cells [25], and hypoxic tumor cells display about a 1.5 times enhanced uptake compared to oxygenated tumor cells [26,27]. Necrotic cells do not consume glucose. Thus, the pSUV values were set to 0, 1, 8, and 12 for necrotic, normal, oxygenated tumor, and hypoxic tumor cells, respectively. Under these assignments, time sequences of cell-level parametric pSUV images (2000 × 2000 pixels, pixel size 0.02 mm) were generated from the sequences of agent maps.

To simulate realistic PET resolution and noise, each cell-level pSUV image was scaled to the units of activity concentration, forward-projected into a sinogram space with realistic added Poisson noise, and reconstructed using ordered-subset expectation maximization (OSEM) with pixel size 0.58 mm and resolution 2.35 mm full width at half maximum (FWHM). The reconstructed image dimensions were 68 × 68 pixels. The forward projection and reconstruction was performed using a previously published and publicly available image reconstruction framework written in Matlab (https://www.bccrc.ca/dept/io-programs/qurit/index.php/software/pet-simulation-and-image-reconstruction accessed on 15 August 2023) [28].

Post-reconstruction image noise was measured using a uniform reference phantom image with pSUV set to 2.37, which modeled the average FDG uptake in the liver [29]. We adjusted the simulation and reconstruction parameters to obtain tumor images with 0% (noise-free), 5%, 10%, and 15% noise, measured in units of normalized standard deviation (NSTD). These noise levels cover the range of noise typically observed in clinical PET scans and measured over the liver [30].

### 2.7. Radiomics Features

For each simulated PET image, we computed all 22 Haralick features that are available in the Pyradiomics framework [31], which is compliant with the Image Biomarker Standardization Initiative (IBSI) [32]. These features included autocorrelation, joint average, cluster prominence, cluster shade, cluster tendency, contrast, correlation, difference average, difference entropy, difference variance, joint energy, joint entropy, IMC1, IMC2, inverse difference moment, inverse difference moment normalized, inverse difference, inverse difference normalized, inverse variance, maximum probability, sum entropy, and sum of squares. The features were computed using the Pyradiomics package (version 3.0.1) in a Python virtual environment (version 3.8.19). The pixel intensities were quantized using the constant bin size technique, as per IBSI and Pyradiomics recommendations, and the bin size was 0.5 (in pSUV units). No image normalization was applied. To compute the gray-level co-occurrence matrix (GLCM), the pixel dimensions were specified to be isotropic, and the GLCM quantification distance was 1 pixel in each dimension. We used a 2.5D direction-merged GLCM. All features were extracted within lesion masks without image re-sampling.

The features were computed every 10 h biological time step to observe the progression of each feature value with tumor growth.

### 2.8. Analysis

First, to validate the model, we analyzed the produced tumor phenotypes and measured the tumor growth rates along with the concentrations of nutrients in the tissue. We compared the observed patterns with real histopathology images from human tumor xenografts known to have different microenvironmental features [22,23].

Next, to determine the optimal features for phenotype discrimination, we performed the two-sample t-test and measured the t-scores between visually similar pairs of phenotypes. This was done for all studied features under varying image noise levels.

To quantitatively assess how well the clusters corresponding to different phenotypes were separated in the feature space, we employed two metrics: (1) the silhouette score (SS), which measures the average similarity of data points to their own clusters; and (2) the Calinski–Harabasz criterion (CHC), which compares between-cluster variance to within-cluster variance. With both metrics, higher values indicate better-formed clusters.

Finally, for all 6 simulated phenotypes, we investigated the changes in radiomics feature values with continuous tumor growth from a single cell to full size of about 1.7 cm and arrived at conclusions with regard to the usefulness of radiomics features at different growth stages.

## 3. Results

### 3.1. Phenotype Analysis

**Figure 2:**
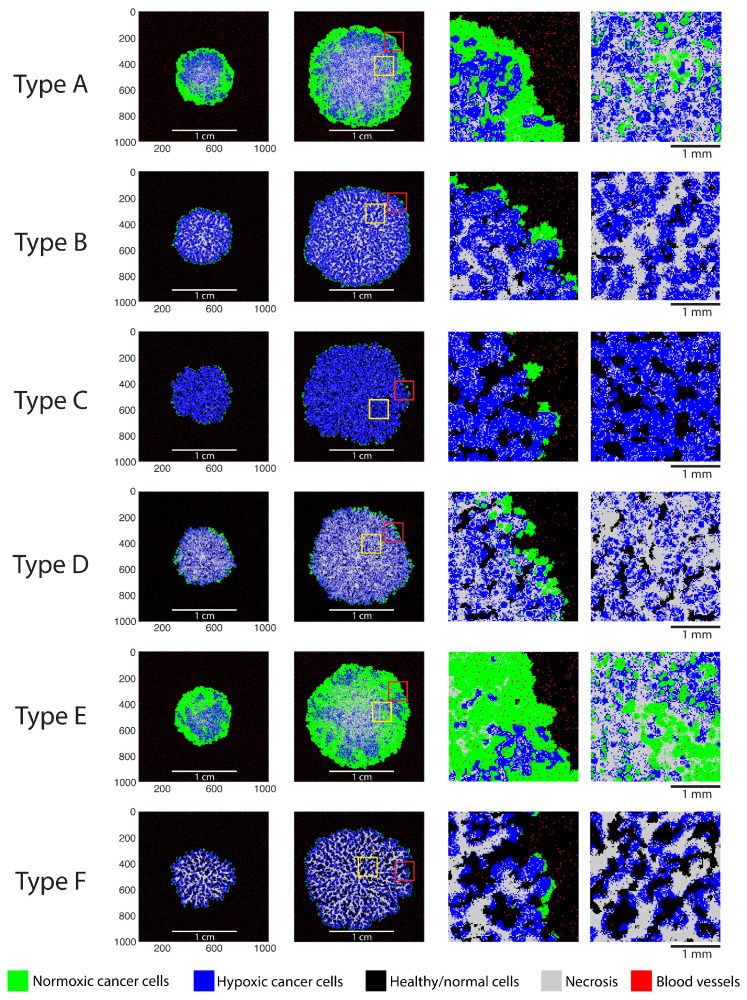
Agent maps illustrating the simulated tumor phenotypes. Columns 1 and 2 contain two longitudinal growth snapshots for each phenotype. Columns 3 and 4 show zoomed-in regions of the agent maps (as indicated by yellow and red squares).

The model was able to generate distinct tumor phenotypes resulting from differences in the tumor microenvironment. The agent maps of simulated tumor phenotypes and their microscopic structures are shown in Figure 2, including two longitudinal snapshots for each phenotype. The simulated phenotypes were visually distinct and manifested a number of features observed in real tumors.

Phenotype A is solid and densely packed (i.e., without healthy tissue pockets) and is characterized by a pronounced necrotic core encompassed by layers of hypoxic and normoxic tumor cells. Most of the blood vessels at the core of this phenotype are replaced by tumor cells, leading to internal necrosis. Phenotype E is similar to phenotype A, with relatively smaller amounts of hypoxia and necrosis, a larger fraction of viable normoxic cells, and a thicker layer of normoxic cells on the periphery. Phenotype D is relatively similar to phenotype E in terms of morphology, except that it contains a larger fraction of necrotic cells. Phenotype B does not present a pronounced core, but rather, necrotic cells are distributed uniformly throughout the tumor. Hypoxic and necrotic cells are present in approximately equal proportions, and the peripheral layer of normoxic cells is relatively thin.

Phenotype C is relatively diffuse and hypoxic, and it incorporates a high amount of healthy tissue. This tumor phenotype exhibits tendril-like structures, which encapsulate pockets of healthy cells as the tumor grows. Phenotype F likewise exhibits tendril-like structures that primarily consist of necrotic cells. Large pockets of healthy tissues are formed, surrounded by hypoxic tumor cells. This was the slowest-growing tumor, and the external layer of normoxic proliferating cells was the smallest among all phenotypes. Overall, Figure 2 demonstrates that the model is capable of producing a wide range of microscopic tumor phenotypes in response to changes in the initial conditions and/or model parameters.

### 3.2. Comparison to Histology Images

**Figure 3:**
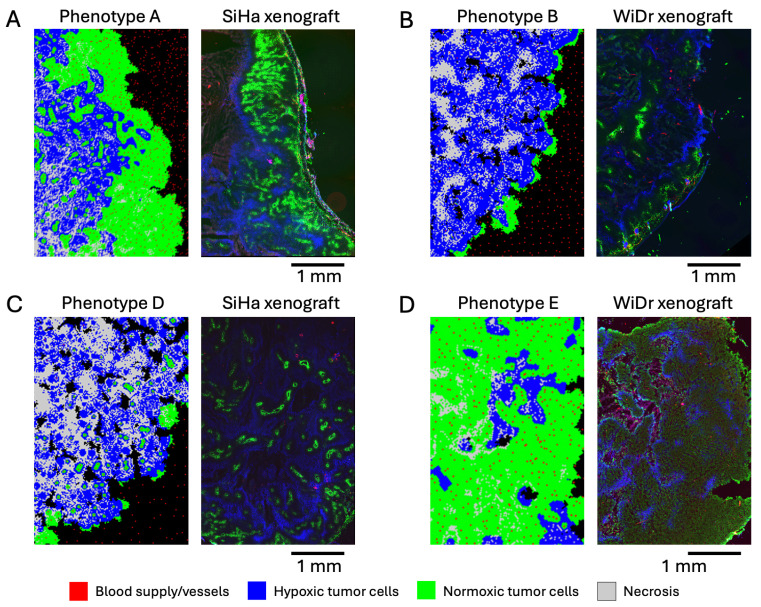
Simulated tumor phenotypes compared to real microscopic images of subcutaneous tumor xenografts. (**A**) Phenotype A compared to SiHa human cervical squamous cell carcinoma. (**B**) Phenotype B compared to WiDr human colorectal adenocarcinoma. The darkest regions within the tumor xenograft show necrosis. (**C**) Phenotype D compared to SiHa human cervical squamous cell carcinoma. (**D**) Phenotype E compared to WiDr human colorectal adenocarcinoma.

To qualitatively evaluate the realism of simulated phenotypes, we compared the simulated agent patterns to real histology slides obtained from subcutaneous tumor xenografts of well-known cell lines (Figure 3).

The normoxic clusters observed in phenotype A around blood vessels can be seen in human cervical squamous cell carcinoma, SiHa line (Figure 3A). The green color in the figure represents regions with active perfusion, meaning normoxic regions with adequate nutrient supply (imaged with Hoechst 33342). The blue color represents regions with hypoxia (imaged with pimonidazole). Note the similarities in the spatial distributions of normoxic clusters and their sizes. Necrotic regions also appear present in the slide (dark blue color), albeit in lesser quantity compared to the simulation. The normoxic cluster sizes are consistent with Thomlinson’s results, where necrosis was triggered in cells that were further than 160 µm from a vessel [33], or about eight cells in our simulation. Another notable feature that appears in the simulated and real images is the hypoxic and normoxic layers on the tumor periphery; the normoxic layer has similar thickness in the simulated and real images.

A thinner normoxic layer on the periphery is present in phenotype B and in the WiDr human colorectal adenocarcinoma line (Figure 3B). In this figure, the green color represents perfusion (imaged with Hoechst 33342), while the blue color indicates hypoxia (imaged with pimonidazole). Small necrotic regions are present on the inner sides of the simulated and real tumors. Interestingly, hypoxic regions in simulations and real images manifest in similar wave-like patterns.

Phenotype D consists mostly of necrotic, hypoxic, and healthy cell clusters, with a few normoxic regions. A very similar pattern (both in terms of size and distribution of the clusters) can be observed in human cervical squamous cell carcinoma (Figure 3C). Pockets of healthy tissue found in phenotypes B and D also appear in the real histological images of solid tumors, although they are not visible in the shown histology slides.

The dense pattern of normoxic and hypoxic cells without necrosis in phenotype E can also be observed in the real image of squamous cell carcinoma (Figure 3D).

To summarize, the cellular patterns that we observed in simulated phenotypes also appear in the real histology slides of tumors, which indicates the realism of the model.

### 3.3. Quantitative Model Validation

For quantitative validation of the model, we compared the macroscopic uncontrolled model observables, such as tumor growth rates and steady-state nutrient concentrations, to real experimental data. The nutrient concentrations in steady state were achieved entirely through diffusion processes in the PDE grids and were not predetermined prior to the simulation. Thus, they represent the derivative measures of the simulations that can be used for model validation.

The approximate time required to reach ∼1 cm in diameter for tumors was 96 days for phenotype A, 158 days for phenotype B, 180 days for phenotype C, 258 days for phenotype D, 108 days for phenotype E, and 196 days for phenotype F. The mean time to reach a 1 cm diameter for all phenotypes was 166 ± 60 days, which is a realistic growth rate. The tumor diameter increase was linear with time.

The average oxygen and glucose concentrations within normal and tumor tissue were measured at the end of each simulation. The average oxygen concentration in normal tissue was 50 mmHg, whereas in the tumor tissue, the concentration was approximately 6.8 ± 4.4 mmHg. Glucose concentrations were also computed, and the average values were 4.7 mM for normal tissue and 1.3 ± 0.6 mM for tumor tissue, which falls within the normal range of glucose concentrations [34,35,36].

### 3.4. Simulated PET Images

**Figure 4:**
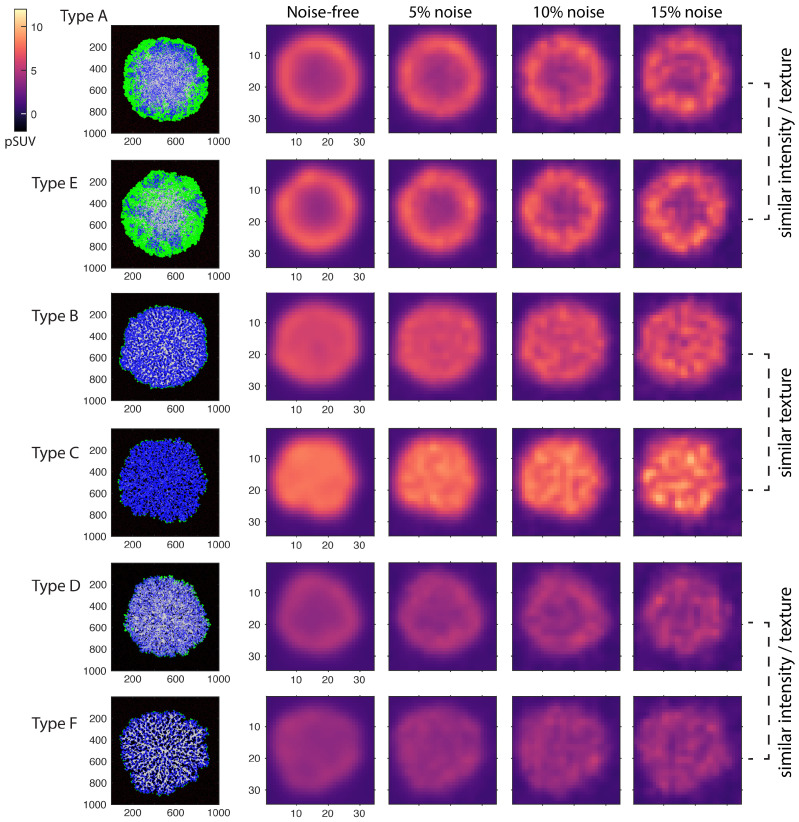
Synthetic PET images produced for the simulated tumor phenotypes, noise free and with 5%, 10%, and 15% image noise levels. The agent grids were converted to images of expected FDG uptake, forward-projected into sinogram space with added Poisson noise, and reconstructed with a resolution of 2.4 mm FWHM. Noise was regulated by adjusting the simulated acquisition time.

Examples of noise-free and noisy PET images generated using the simulated agent maps are plotted in Figure 4. Noise levels of 5% and 15% NSTD represent the minimum and maximum noise typically found in routine imaging, while 10% NSTD represents the average noise level [30]. The figure demonstrates that the simulated PET images, particularly noise-free and at low 5% noise, preserve spatial features that allow for phenotype differentiation. Images of phenotypes B, C, and F manifest inner-tumor heterogeneity even at a resolution that is 100 lower than the agent grid size (i.e., 2.35 mm FWHM vs. 0.02 mm cell size). The phenotypes can also be visually identified by the tumor intensity, although in reality, this would be confounded by a greater-than-simulated variability in the tracer uptake.

As expected, in PET images, phenotype differentiation becomes more difficult with higher noise. For example, the necrotic core in phenotype D is visible in noise-free and 5% noise images and is obfuscated at 10% and especially 15% noise. Likewise, phenotypes A and E become virtually indistinguishable at 10% and 15% noise. Better identification of such phenotypes in practice may guide therapy decisions. For example, A/E phenotype differentiation can be linked to the task of identifying more aggressive tumors, and D/F phenotype differentiation can be linked to identifying solid/diffuse tumors.

In the next section, as an example application of our method, we identify radiomics features that can best differentiate all six simulated phenotypes. In addition, we describe the analysis of the longitudinal tumor growth to understand at what tumor sizes the chosen features remain informative.

### 3.5. Radiomics Analysis

We analyzed all 22 Haralick texture features available in the Pyradiomics library. For each feature, we ran a t-test between two distributions of feature values corresponding to two chosen phenotypes. The resulting t-scores for features computed at 10% image noise are given in Table 3. The data demonstrate that different features were optimal for the discrimination of different phenotype pairs. For example, inverse difference worked reasonably well for discriminating all pairs except D/F. On the contrary, inverse difference moment normalized was only effective in differentiating E/C but not other phenotypes. The most difficult phenotypes to differentiate were D/F, followed by A/E; this is consistent with their similar visual appearance both in terms of texture and intensity (Figure 4). Notably, no single feature was able to provide a statistically significant discrimination between D and F phenotypes with 10% image noise or higher.

In the rest of the analysis, we selected a subset of features that had t-scores greater than 3.0 for either A / E phenotype pair or D / F pair. The t-scores for this subset of features measured with 5% and 15% image noise are given in Table 4. The magnitude of image noise was found to have a strong and variable impact on discrimination performance. For example, per Table 4, with 5% image noise, phenotypes E and C can be easily discriminated by almost any feature. On the other hand, with 15% noise, only cluster shade and difference variance provide significant discrimination. In comparison, phenotypes A and E can be differentiated in 5% and 15% noisy images by the same set of features; specifically, cluster shade, cluster tendency, inv. difference moment, inverse difference, and sum of squares maintain significant (albeit slightly lower) t-scores with 15% noise. Across all phenotype pairs, the features of inverse difference normalized and inverse variance were particularly sensitive to noise since they did not provide significant discrimination between any phenotype pair in 15% noise images.

These observations demonstrate that features vary greatly in terms of their discriminative ability and sensitivity to image noise. It follows that it should be possible to find optimal features for phenotype analysis that are more noise resilient than are others.

The data in Table 3 and Table 4 demonstrate that no single feature was able to differentiate between all six phenotypes. However, a combination of features can be selected to uniquely classify all six of the simulated phenotypes with relatively high accuracy. To differentiate phenotypes D and F, between difference entropy and difference variance, we chose the former since it had higher overall t-scores. To differentiate between other phenotype pairs, we chose cluster shade; however, cluster tendency is a close alternative. Cluster shade and cluster tendency can discriminate between most phenotypes while being relatively resilient to noise.

**Figure 5:**
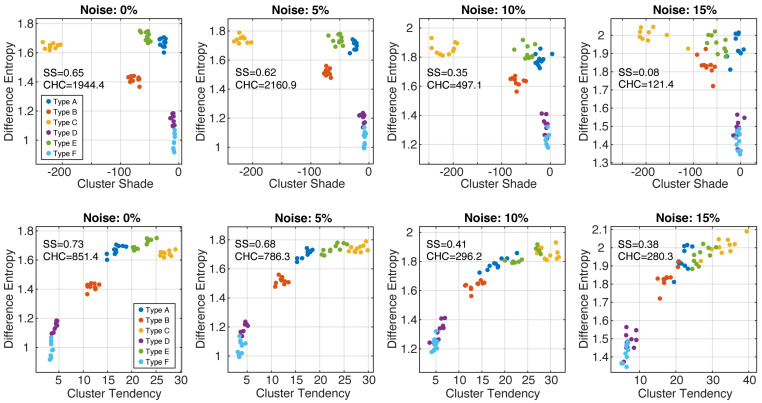
Together, GLCM difference entropy and GLCM cluster shade (**top row**) were able to identify all six phenotypes with image noise up to 15%. The pairs GLCM difference entropy and GLCM cluster tendency (**bottom row**) represent a good alternative. SS—silhouette score; CHC—Calinski–Harabasz criterion.

Figure 5 shows the scatter plots of the feature values (difference entropy, cluster shade, cluster tendency) for all phenotypes at different noise levels (data from two sequential growth steps are shown for better visualization). In noise-free and 5% scatter plots, all six phenotypes formed separable clusters. With higher noise levels, the clusters became less defined, and robust phenotypes classification became problematic. This observation is reflected in the measured values of SS and CHC, which both decreased with higher image noise. The greatest reduction in both clustering metrics occurred when going from 5% noise to 10% noise. The scatter plots also demonstrate that noise introduced bias in the feature values; in particular, difference entropy values increased with higher noise.

Overall, we found that a combination of difference entropy and cluster shade (or cluster tendency) was optimal to providing an accurate identification of all six phenotypes at various noise levels.

### 3.6. Longitudinal Analysis

**Figure 6:**
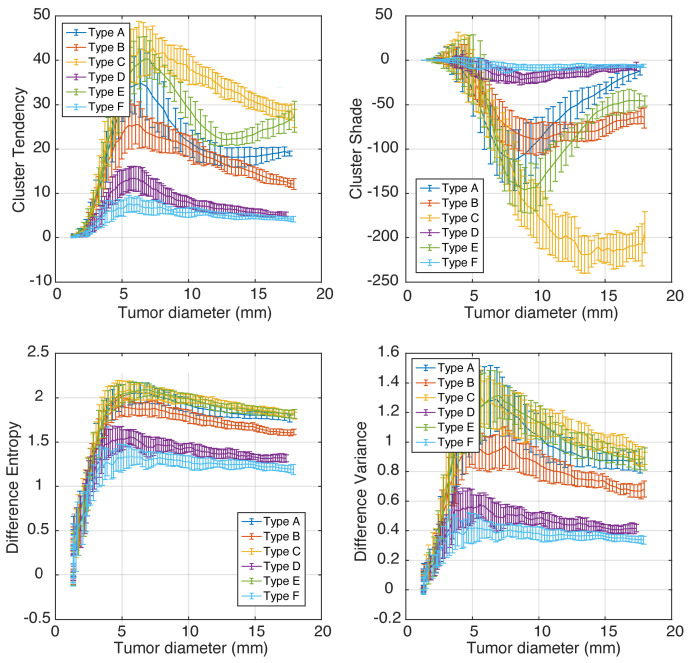
A representative set of Haralick features plotted as functions of tumor size (longitudinal tumor growth).

Longitudinal simulations of tumor growth can help estimate the minimum tumor size requirements for phenotype classification and measure how different radiomics features change with tumor progression. The graphs of cluster tendency, cluster shade, difference entropy, and difference variance versus tumor diameter are plotted in Figure 6. The error bars encapsulate variances from different phenotype realizations (N = 5) and from image noise.

Importantly, we observed that some features changed non-linearly and non-monotonically with tumor growth; e.g., when tumors grow larger than 5 mm, difference entropy and difference variance changed monotonically, while cluster tendency and cluster shade had a secondary extrema for some phenotypes. This behavior can confound radiomics-based studies that include multiple tumor diameters and rely on linear modeling. Consider, for example, the values of cluster tendency for phenotype E: the feature values will be similar for tumor diameters 5 mm, 11 mm, and 17 mm due to the “N”-like shape of the graph.

On the other hand, the values of cluster shade for phenotypes A, B, and D changed relatively little with tumor diameter, which makes them a poor choice for tracking tumor growth or response to therapy. The best features for tumor response and/or progression tracking are those that increase monotonically and have small error bars—e.g., difference entropy.

We finally notice that for tumor diameters under 5–9 mm, there was significant overlap of feature values corresponding to different phenotypes, although this was feature-dependent. Difference entropy can be informative for sizes above ∼5 mm, and cluster Shade was only discriminative for tumors larger than ∼9 mm. In our simulations, 9 mm is equivalent to 3.8 resolution units.

## 4. Discussion

We developed a new method to simulate PET images of biologically plausible tumor phenotypes and their longitudinal development. The proposed method can be used to investigate the interplay between the values of PET-derived radiomics features and tumors’ intrinsic and extrinsic properties, such as nutrient supply and vasculature. Furthermore, the influence of image noise, resolution, and other imaging factors on the descriptive strength of features can be investigated.

In comparison to our approach, the static radiomics phantoms used in previous studies have several downsides: (1) they do not allow for longitudinal studies and lack the temporal dimension, (2) they have simplified geometries that are not reflective of real tumor complexity, (3) they do not provide the means of generating subtle variations of morphology and keep the overall patterns the same between different phantom realizations, and (4) they are not linked to the fundamental underlying biology or microenvironmental factors. In contrast, we believe that the proposed methodology will enable new investigations that include modeling of multiple factors such as tumor environment, phenotype, image noise, resolution, and region-of-interest definitions. It may also guide the development of new features that are informative, robust, and reliable, ultimately allowing for more optimized treatment planning and prognosis.

As an example application of the new method, we performed an optimal-feature search for the differentiation of phenotypes containing various quantities of normal, hypoxic, and necrotic cancer cells in the presence of image noise. Distinct phenotypes were obtained by modifying the blood vessel density, vessel removal probability, vessel distribution pattern, and cell death probability from the depletion of nutrients. We demonstrated that not a single feature among the 22 tested could differentiate between all six phenotypes at realistic noise levels, and at least two features were needed for accurate phenotype classification. This finding highlights the importance of multivariate analysis. Standardized Haralick features of difference entropy and cluster shade from the widely-used PyRadiomics framework were found to be the most sensitive and noise resilient for the differentiation between and classification of the simulated phenotypes. Our results suggest that radiomics analysis can be beneficial even in small lesions with a diameter of 3.5–4 resolution units. Another important finding is that feature values may change non-monotonically with tumor growth, which may render certain features non-suitable for tracking disease progression or therapy response.

Several model extensions are possible that can enable new directions of studies. The model parameters and initial conditions can be adjusted to simulate countless scenarios, for instance, by using different cancer cell types and incorporating other biomolecules that influence tumor growth. Due to the hybrid nature of the model, different types of agents and PDE grids can be easily incorporated. Of particular interest may be the incorporation of a PDE grid for chemotherapy drugs or radiopharmaceutical molecules to investigate the absorbed dose and effectiveness of radiopharmaceutical therapy in different tumor types. For the addition of each new molecule, a new PDE grid should be added to the model, and the diffusion coefficient in tissue should be specified. The parameters of the agents used in the model can also be adjusted within the biologically reasonable range to simulate different cell lines and genetic variations. Cancer hallmarks such as angiogenesis and clearing of necrotic regions can be modeled as well. This can be achieved by entering additional functions in a cell decision step to determine whether a cell will create new vessels or replace necrotic cells with normal non-cancerous cells.

Another extension can be the addition of intra-tumor genetic variability and environmental pH. Both factors are known to play an important role in tumor resistance to chemotherapy and radiotherapy [37]. In fact, in a previous study conducted by Robertson-Tessi et al., the authors demonstrated the use of a hybrid mathematical oncology modeling approach by simulating tumor growth in vascularized tissue to assess the effects of tumor microenvironments on tumor phenotypic adaptations [8]. The cells in the model had a continuously variable metabolic phenotype and different resistance to acidic environments, which are both traits that are passed on from the parent to the daughter cells with small variations. Similarly to our approach, point source vasculature was used to represent the vessels crossing the 2D plane delivering nutrients to the tissue. The vessel distribution was altered through angiogenesis or vessel degradation. The effects of the non-uniform microenvironment on acidosis and the development of aggressive traits in acid-resistant cells have been examined.

The basic mechanism of our tumor growth model is similar to that of Robertson-Tessi et al.’s; however, the central focus here is to link the observable tumor heterogeneity in synthetic PET images to the tumor tissue microenvironment, evaluated using a quantitative radiomics approach. Thus, it should be relatively straightforward to incorporate environmental acidity and genetic variability within our method.

A major limitation of our approach is that it does not include the modeling of mechanical forces, such as cell cohesion, tissue resistance to tumor expansion, or blood vessel elasticity. Hence, our present model does not include the effects of shear stress and external pressure; the validity of our current simulations is thus restricted to small tumors embedded in uniform tissue, where the external forces on the growing tumors are expected to be small. In future models, these effects can be accounted for by introducing and calculating forces at each grid location. A new parameter can be introduced in the cell division probability that depends on the forces at the cell’s location as well as the composition of the neighboring agents on the grid. Thus, the force field will influence the rate and direction of tumor cell proliferation.

Another limitation is that our method has only been tested in 2D. The hybrid automata library offers an option to extend the simulations to a 3D grid, which may be a more realistic model of volumetric cancer (albeit much more computationally expensive). The purpose of the simulation in this study is to assess tumor heterogeneity arising from metabolic heterogeneity and variations in the tumor microenvironments, so a 2D slice representing a cross-section of the solid tumor was deemed to be sufficient.

Future work will focus on generating and testing additional phenotypes and on further improvement and validation of the model’s realism. A “standard database” of phenotypes can be created to be used in PET radiomics studies as advanced phantoms. Another future research direction is the addition of radiopharmaceutical compounds to the simulation, which will enable the investigation of absorbed doses to cells in different phenotypes [38].

## 5. Conclusions

We developed a novel method and model to simulate realistic tumor phenotypes on a microscopic level and their corresponding macroscopic PET images. Starting from a single cell, the model can simulate longitudinal tumor development with realistic tissue morphology, tumor growth rates, and nutrient concentrations. Using the proposed method, we showed that standardized radiomics features can discriminate between visually similar microscopic phenotypes of relatively small tumors in noisy PET images. We showed that different features were optimal for discriminating different pairs of phenotypes, highlighting the importance of multivariate analysis. Haralick’s difference entropy, cluster shade, and cluster tendency were identified as the most discriminative and noise-resilient features. Furthermore, we showed that features may change non-monotonically with tumor progression, with has important implications for feature selection in tracking disease progression or therapy response. In future work, the developed method can help guide the development of new features that are informative, robust, and reliable, ultimately allowing for more optimized treatment planning and prognosis.

## Figures and Tables

**Table 1:** Model biological parameters that were constant in all simulated phenotypes.

Biological Constant	Values
Average cell (unit) size	20 µm
Average time for aerobic cell division	24 h
Vessel density range	20–100 mm^2^ [14,15,16]
Oxygen diffusion coefficient in tissue	$1.65\times 10^{-5}$ cm^2^/s [17]
Glucose diffusion coefficient in tissue	$2.7\times 10^{-6}$ cm^2^/s [18]
Oxygen concentration in blood vessel	50 mmHg
Glucose concentration in blood vessel	$5\times 10^{-3}$ mol/L
Oxygen consumption rate—normal tumor	$4\times 10^{-15}$ mol/min/cell [19]
Oxygen consumption rate—hypoxic tumor	$2\times 10^{-15}$ mol/min/cell [20]
Oxygen consumption rate—non-cancer cell	$2.5\times 10^{-18}$ mol/min/cell [21]
Glucose $V_{max}$—normal tumor	$5\times 10^{-14}$ mol/min/cell
Glucose $V_{max}$—hypoxic tumor	$1.02\times 10^{-13}$ mol/min/cell
Glucose $V_{max}$—non-cancer cell	$5\times 10^{-15}$ mol/min/cell
Glucose $K_{M}$	$2\times 10^{-14}$ mol/min/cell
Death ATP production rate	$2.57\times 10^{-14}$ mol/min/cell

**Table 2:** Model variable parameters specific to each tumor phenotype.

Phenotype	Vessel Density [mm^−2^]	Vessel Removal Probability	Vessel Distribution Pattern	Necrotic Death Probability (ATP)
Type A	100	0.05	Random	$1-R^{2}$
Type B	50	0.05	Uniform density	$1-R^{2}$
Type C	50	0.5	Random	1−R
Type D	20	0.05	Uniform density	$1-R^{2}$
Type E	80	0.02	Random	1−R
Type F	50	1	Random	$1-R^{2}$

**Table 3:** t-scores for discrimination between pairs of phenotypes. Rows correspond to different Haralick features, and columns represent phenotype pairs. The features were computed from PET images with 10% noise. Values with p<0.01 are highlighted in bold.

	A vs. E	C vs. B	D vs. F	A vs. B	E vs. C	E vs. B
Autocorrelation	0.22	**9.07**	1.41	0.60	**8.96**	0.34
Joint Average	0.01	**8.14**	1.23	0.86	**8.76**	0.80
Cluster Prominence	2.90	**23.49**	2.56	0.99	**13.72**	**3.92**
Cluster Shade	**3.79**	**21.87**	1.80	**6.49**	**19.67**	1.69
Cluster Tendency	3.18	**24.63**	2.24	**5.12**	4.15	**8.52**
Contrast	1.86	**11.61**	2.79	**7.13**	0.50	**8.95**
Correlation	**4.42**	**7.49**	0.01	1.50	**7.99**	**4.07**
Difference Average	2.83	**10.84**	2.35	**8.76**	2.15	**10.58**
Difference Entropy	1.17	**10.63**	3.38	**6.52**	0.81	**8.14**
Difference Variance	0.17	**9.00**	3.35	**5.11**	2.52	**6.37**
Joint Energy	2.42	**7.57**	1.77	**18.39**	**7.80**	**20.39**
Joint Entropy	2.09	**11.18**	2.14	**10.85**	**5.12**	**13.62**
IMC1	0.14	**5.27**	0.88	0.92	**8.15**	1.07
IMC2	1.51	**8.51**	0.10	**5.03**	3.65	**6.70**
Inv. Diff. Moment	**3.57**	**8.71**	2.15	**9.63**	3.30	**11.33**
Inv. Diff. Moment Norm.	2.50	1.83	0.97	0.52	**6.36**	2.48
Inverse Diff.	**3.84**	**8.64**	1.99	**9.82**	**3.72**	**11.99**
Inverse Diff. Norm.	**4.27**	0.74	1.26	2.03	**7.91**	**5.35**
Inverse Variance	**6.38**	0.55	0.90	2.39	**15.95**	**7.02**
Maximum Probability	1.32	**4.36**	1.35	**27.05**	**6.52**	**15.19**
Sum Entropy	2.23	**13.66**	1.73	**10.47**	**4.38**	**13.65**
Sum Squares	3.12	**24.54**	2.35	**5.29**	3.87	**8.64**

**Table 4:** t-scores for discrimination between pairs of phenotypes. Rows correspond to different Haralick features, and columns represent phenotype pairs. The features were computed from PET images with 5% and 15% noise. Values with p<0.01 are highlighted in bold.

	Image Noise 5%					
	**A vs. E**	**C vs. B**	**D vs. F**	**A vs. B**	**E vs. C**	**E vs. B**
Cluster Shade	**6.47**	**25.32**	**2.64**	**20.04**	**23.62**	**3.53**
Cluster Tendency	**4.64**	**14.74**	**3.57**	**7.38**	**4.07**	**9.74**
Correlation	**5.58**	**8.26**	**0.03**	**0.04**	**7.06**	**3.17**
Difference Entropy	1.86	**16.95**	**5.20**	**13.21**	**0.25**	**12.71**
Difference Variance	**0.19**	**13.22**	**5.31**	**9.55**	**4.58**	**7.91**
Inv. Diff. Moment	**4.20**	**7.25**	3.20	**8.99**	**6.16**	**15.86**
Inverse Diff.	**4.37**	**6.69**	2.85	**8.91**	**6.89**	**16.21**
Inverse Diff. Norm.	**1.42**	0.91	1.09	**4.16**	**5.81**	**6.21**
Inverse Variance	**4.04**	**1.72**	**0.99**	**6.27**	**10.09**	**9.97**
Sum Squares	**4.59**	**15.10**	**3.79**	**7.75**	**3.90**	**10.03**
	**Image Noise 15%**					
	**A vs. E**	**C vs. B**	**D vs. F**	**A vs. B**	**E vs. C**	**E vs. B**
Cluster Shade	**5.07**	**15.62**	0.23	**9.50**	**14.75**	2.07
Cluster Tendency	**4.87**	**9.90**	1.68	**6.42**	3.15	**10.18**
Correlation	3.32	**7.72**	0.86	2.45	2.85	**5.44**
Difference Entropy	1.49	**9.91**	**3.74**	2.71	3.32	**11.01**
Difference Variance	0.76	**12.45**	**3.62**	1.99	**5.29**	**5.55**
Inv. Diff. Moment	**3.95**	**6.04**	2.62	**6.51**	0.01	**7.80**
Inverse Diff.	**4.17**	**6.15**	2.40	**6.36**	0.11	**7.96**
Inverse Diff. Norm.	0.52	0.09	0.70	1.68	1.94	2.00
Inverse Variance	0.56	2.12	1.88	0.25	2.79	0.25
Sum Squares	**4.74**	**9.87**	1.98	**6.27**	3.13	**10.42**

## Data Availability

The raw data supporting the conclusions of this article will be made available by the authors on request.
